# Optical Fiber Time Delay Comparison Between NIST and LAMETRO

**DOI:** 10.6028/jres.126.040

**Published:** 2022-01-10

**Authors:** T. Dennis, J. Jimenez

**Affiliations:** 1 National Institute of Standards and Technology, Boulder, CO 80305, USA; 2 Laboratorio de Metrología (LAMETRO), Instituto Costarricense de Electricidad (ICE), San José, 10032-1000, Costa Rica

**Keywords:** BIPM, communications, international comparison, modulation phase shift, optical fiber, optical time domain reflectometer, OTDR, time delay

## Abstract

We describe the results of a bilateral measurement comparison of optical fiber time delay between the National Institute of Standards and Technology (NIST, USA) and Laboratorio de Metrología, Instituto Costarricense de Electricidad (LAMETRO-ICE, Costa Rica), which was conducted on a single-mode optical fiber reference spool at wavelengths of 1310 nm and 1550 nm. The measurement results showed the largest difference to be less than 0.93 ns, which is within the combined standard uncertainty (coverage factor k = 1) for the measurement systems at the two laboratories.

## 1 Introduction

To demonstrate and maintain their technical competence as well as quality system compliance, national metrology institutes such as the Laboratorio de Metrología (LAMETRO, Costa Rica) and the National Institute of Standards and Technology (NIST, USA) periodically undertake comparisons of their measurement systems and protocols. Publication of such comparisons is an important part of maintaining standing with the Bureau International de Poids et Mesures (BIPM), which is served by regional metrology organizations such as the Inter-American Metrology System (SIM), of which LAMETRO and NIST are members. Construction and maintenance of optical communications infrastructure underpin the telephony and high-speed networking (*i.e.*, World Wide Web) of modern life. Optical fiber–based communication relies on accurate optical fiber time delay measurements to determine accurate optical lengths of different elements within the optical network to reduce the cost of infrastructure maintenance for public and private users.

The aim of this project was to perform a comparison of methods for the measurement of time delay of a single-mode optical fiber spool at wavelengths of 1310 nm and 1550 nm. The comparison was made by measuring the time delay of a reference spool consisting of type G.652 optical fiber approximately 10 km in length. Type G.652 optical fiber is designed to be low loss and single mode at 1310 nm and 1550 nm for use in optical communications systems. Specifically, model SMF-28 fiber manufactured by Corning Incorporated is readily available and was used in this comparison.^1^1 Certain commercial materials are identified in this paper to specify the experimental apparatus accurately. Such identification does not imply recommendation or endorsement by the National Institute of Standards and Technology, nor does it imply that the materials or equipment identified are necessarily the best available for the purpose.

The fiber optic spool was supplied and packaged by NIST. NIST measured the time delay of the spool first and then sent it to LAMETRO for them to perform their measurements. LAMETRO measured the time delay for the spool at both wavelengths and submitted these values to NIST at the conclusion of their measurements.

For time delay measurements at NIST, the primary standard for traceability is a hydrogen maser maintained at NIST that provides a representation of the SI unit of time, the second. In the NIST measurement system described below, a vector network analyzer locked to the hydrogen maser (standard uncertainty [*k* = 1] on the order of 1 × 10^-14^ s) is used to transfer the unit of time to delay measurements. The notation “*k* = 1” is used to denote a coverage factor for uncertainty that defines an approximate 65% confidence interval, whereas “*k* = 2” denotes an expanded uncertainty with an approximate 95% confidence interval [1]. The reference standard for traceability at LAMETRO is a fiber optic spool artifact, which was previously calibrated by the National Metrology Institute of Mexico (CENAM) using the frequency-domain phase-shift technique described in Ref. [2] with an expanded uncertainty (*k* = 2) of 3.1 µs/s for delay. Like NIST, traceability for the CENAM spool artifact calibration is also provided by a hydrogen maser that realizes the SI unit of time. In this comparison of fiber time delay, NIST has direct traceability to the SI unit of time, while LAMETRO relies on a transfer process for traceability based on a reference standard.

## 2 Transfer Standard

**Fig. 1:**
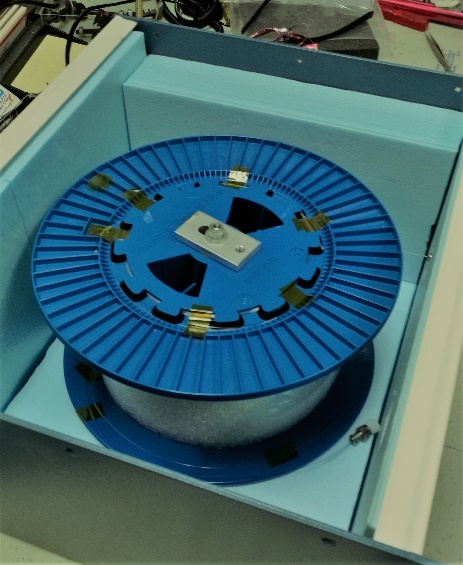

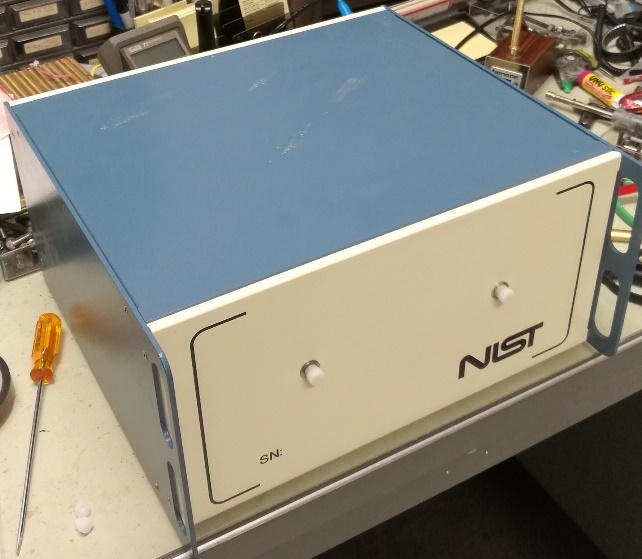
Photographs of the NIST-built reference spool containing an approximately 10 km length of G.652 single-mode optical fiber.

To facilitate this comparison, we used a NIST-built transfer standard, which consisted of a G.652 fiber optic spool packaged in a metal case designed to hold the fiber securely and safely as shown in Fig. 1. Two patch cables with an approximate length of 0.1 m and fitted with ferrule connector/angle physical contact (FC/APC) connectors were fusion-spliced to both ends of the approximately 10 km fiber spool. To reduce the impact of temperature and humidity, the inside of the metal case was filled with expanding foam as a “potting” material to seal it off. The outside of the fiber windings of the spool were wrapped in soft plastic padding to avoid direct contact with the foam, which was applied with the lid off to allow the foam to expand freely while reducing strain on the fiber. When used in a temperature-controlled oven, the change in fiber length caused by temperature-dependent strain was found to be negligible.

The transfer standard, also referred to as the “device under test” (DUT), was calibrated at both national laboratories using their respective measurement systems, which are traceable to the fundamental SI unit of time.

## 3 Measurement Systems

**Fig. 2:**
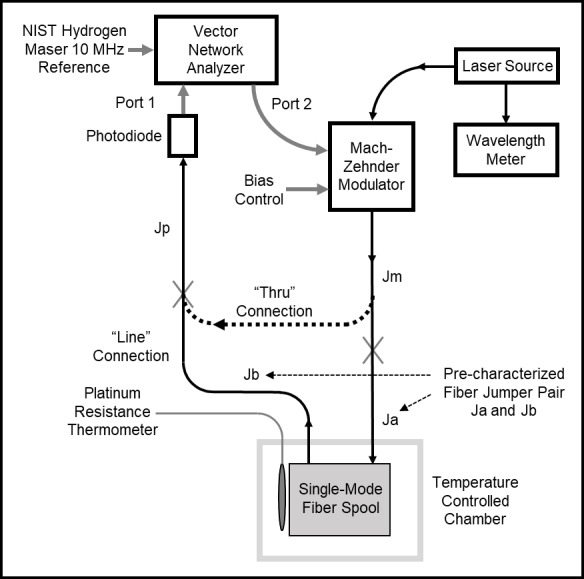
NIST measurement system. Thin black lines represent optical paths, while thick gray lines represent electrical paths. The gray “X” symbols represent FC/APC connections that are changed manually during the calibration process. The fiber cable Jp connected to the photodiode can be connected directly to the Mach-Zehnder modulator jumper Jm for a “thru” connection or to the jumper Jb from the fiber spool for the “line” connection. The temperature of the spool is stabilized in a temperature-controlled chamber. The chamber has a jumper pair (denoted Ja and Jb) to connect the fiber spool to the measurement system.

The NIST measurement system is depicted in Fig. 2. The propagation time delay of the fiber spool was measured using a frequency-domain phase-shift technique based on the method described in several references [2–4]. In short, the phase delay caused by propagation of an amplitude-modulated optical signal through the spool was measured as a function of modulation frequency. The time delay of the spool was found by iteratively fitting the measured phase to increasingly accurate estimates of the time delay as a function of frequency.

**Fig. 3:**
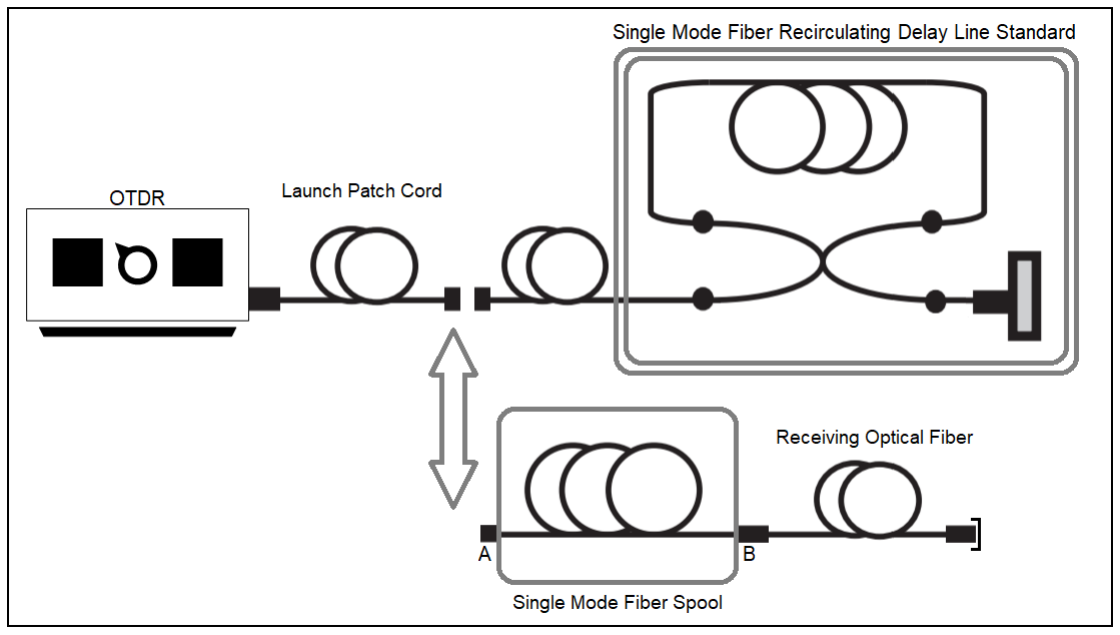
LAMETRO measurement system to realize a pulse propagation delay measurement with an optical time delay reflectometer (OTDR). The delay line standard was previously calibrated at a national metrology institute external to LAMETRO.

The LAMETRO measurement system is depicted in Fig. 3. The value of optical time of flight of the single-mode fiber spool was determined by the pulse propagation delay method [5] using an optical time domain reflectometer (OTDR) calibrated through a single-mode fiber recirculating delay line standard. LAMETRO’s fiber spool reference standard is traceable to the SI unit of time as previously provided by CENAM. Because the OTDR at LAMETRO only reports a distance measurement, optical fiber time delay was determined by assuming a propagation index of 1.46, consistent with the constant assumed in the calibration provided by CENAM.

## 4 Results of the Comparison

The NIST and LAMETRO measurement capabilities were compared by means of the measured time delay of the transfer standard in units of nanoseconds (ns), at the nominal vacuum wavelengths of 1310 nm and 1550 nm and a spool temperature of 20 °C. In performing the measurements, the laboratories followed the guidelines and procedures described in the International Electrotechnical Commission (IEC) standards 60793-1-22 [3] and 61746-1 [5]. The uncertainties for the time delay measurements were evaluated in accordance with the International Organization for Standardization (ISO) document standard [1].

A summary of results for the comparison of measured optical fiber time delay by the two participants is given in Table 1. At 1310 nm, the difference between the NIST and LAMETRO results was only −0.037 ns, while at 1550 nm, the difference was −0.930 ns, where the minus sign for both differences indicates that the LAMETRO measurement system read lower than that of NIST. The NIST standard uncertainty was 0.036 ns at 1310 nm and 0.037 ns at 1550 nm, while that of LAMETRO was 1.5 ns at 1310 nm and 3.2 ns at 1550 nm.

**Table 1:** Comparison of optical fiber delay measurement results between LAMETRO and NIST for a nominal 10 km spool at 20 °C.

Reference Wavelength (nm)	LAMETRO Delay (ns)	NIST Delay (ns)	Delay Difference (ns)	LAMETRO Standard Uncertainty (ns)	NIST Standard Uncertainty (ns)	Combined Standard Uncertainty (ns)
1310	48309.4	48309.437	−0.037	1.5	0.036	1.5
1550	48329.6	48330.530	−0.930	3.2	0.037	3.2

At NIST, repeated measurements of the DUT were conducted, and the standard deviation was taken as an estimate of the uncertainty for repeatability, finding a standard deviation of 0.011 ns at a wavelength of 1310 nm and 0.010 ns at a wavelength of 1550 nm. At LAMETRO, repeated measurements of the DUT were conducted, and the standard deviation was taken as an estimate of the uncertainty for repeatability, finding a standard deviation of 0.57 ns at a wavelength of 1310 nm and 0.81 ns at a wavelength of 1550 nm. Estimates for additional sources of uncertainty for each of the measurement systems are summarized in Tables 2 and 3. For the NIST system, temperature-controlled distributed feedback lasers at 1310 nm and 1550 nm were used, and the wavelength stability of better than 0.01 nm was monitored with a wavelength meter. Numerical compensation of chromatic dispersion for G.652 fiber was applied for the exact reporting wavelengths, with higher uncertainty at 1550.0 nm caused by greater separation from the zero-dispersion wavelength [4]. For the LAMETRO system, the OTDR is a commercial unit that has an event dead zone of 0.5 m and a minimum pulse width of 3 ns. At both wavelengths, the dominant component of uncertainty for the LAMETRO system is the OTDR distance scale calibration, which makes use of the fiber spool reference standard in a recirculating loop (Fig. 3). The large component of uncertainty is a direct consequence of the measurement performance of the OTDR, which has an uncertainty expression given by the manufacturer that depends on a constant, a distance-dependent term, and a resolution-dependent term.

**Table 2:** Measurement uncertainties in nanoseconds for the modulation phase-shift measurement of optical fiber time delay at NIST. VNA stands for “vector network analyzer,” and a coverage factor *k* = 2 defines an interval having a level of confidence of approximately 95%.

NIST Measurement Uncertainties		
Source	Uncertainty (ns)	
	1310.0 nm	1550.0 nm
Type A		
Repeatability	0.011	0.010
Jumper delay	0.000	0.001
Type B		
Wavelength correction	0.005	0.008
Temperature correction	0.034	0.035
VNA dynamic accuracy	0.002	0.002
Combined standard uncertainty	0.036	0.037
Expanded uncertainty (*k* = 2)	0.072	0.074

**Table 3:** Measurement uncertainties in nanoseconds for the pulse propagation delay measurement of optical fiber time delay at LAMETRO. OTDR stands for “optical time domain reflectometer,” and a coverage factor *k* = 2 defines an interval having a level of confidence of approximately 95%.

LAMETRO Measurement Uncertainties		
Source	Uncertainty (ns)	
	1310.0 nm	1550.0 nm
Type A		
Repeatability	0.57	0.81
OTDR calibration	1.1	3.0
Type B		
Launch patch cord	0.014	0.014
OTDR resolution	0.6	0.6
Wavelength correction	0.0	0.14
Temperature correction	0.50	0.50
Combined standard uncertainty	1.5	3.2
Expanded uncertainty (*k* = 2)	3.0	6.4

Table 1 also provides values for the relative combined standard uncertainties between NIST and LAMETRO for this comparison. These values were calculated by taking a square root of the sum of the squares of each laboratory’s standard uncertainties. The observed interlaboratory differences (−0.037 ns at 1310 nm and −0.930 ns at 1550 nm) are less than the relative combined standard uncertainties (*k* = 1) for the measurements by the laboratories.

## 5 Conclusion

The comparison of results presented in Table 1 demonstrates that the differences in measured optical fiber time delays at the two wavelengths between NIST and LAMETRO are both within the combined standard uncertainties of the laboratory measurement systems. This indicates good agreement between the measurement capabilities of the two laboratories despite the use of significantly different methods. However, as a natural consequence of these methods, the standard uncertainties for each of the laboratories (Tables 2 and 3) differ by more than an order of magnitude. As a result, the combined standard uncertainties given in Table 1 are dominated by the uncertainties of the pulse propagation delay method employed by LAMETRO. Because the LAMETRO uncertainties are dominated by the performance of the commercial OTDR equipment, more detailed comparisons, such as measurement wavelength, are difficult to assess. However, based on the respective standard uncertainties, this comparison has provided independent validation of LAMETRO’s measurement capabilities for optical fiber time delay metrology. At the same time, the comparison is a valuable check on the method employed by NIST and illustrates the value that would be gained from a direct intercomparison with another national metrology institute, such as CENAM, which also uses the modulation phase-shift method.
